# A Treatise on Orthopedic Surgery

**Published:** 1907-04

**Authors:** 


**A Treatise on Orthopedic Surgery.** By **Royal Whitman,** M.D., Instructor in Orthopedic Surgery in the College of Physicians and Surgeons, New York; Chief of Orthopedic Department in Vanderbilt Clinic, New York. Third edition, revised and enlarged. Octavo, 900 pages, with 554 illustrations, mostly original. Lea Brothers & Co., Philadelphia and New York, 1907. (Cloth, $5.50 net).

The third edition of Whitman's popular work, shows careful revision and amendment to date, much new material and many new illustrations having been added. In the discussion of Pott's disease new methods are detailed for the application of the plaster jacket in recumbency, and greater stress is laid on the prevention of deformity and the necessity for fixation and traction in paraplegia. Reference is made to Goldthwait's work on the sacro-iliac joints but the discussion is incomplete. The *x*-ray, tuberculin, Bier's treatment, and the Mosetig bone plug are now included. In lateral curvature of the spine the forcible correction, as advocated by Wullstein and Lovett, is added; for the curvature due to anterior poliomyelitis Whitman now says that support must be used. Wolff's law, atrophy of bone due to disuse, and cervical ribs are enlarged upon. The chapter on arthritis deformans is rewritten and Goldthwait's classification is followed. Murphy's method of treating ankylosis is given preference; also operative repair after obstetrical injury to the brachial plexus. Whitman has largely changed the chapter on congenital hip dislocation, and has added his new treatment of fracture of the hip by fixation in abduction. His original work on the foot has enabled him to write most instructive and entertaining descriptions of the deformities of the feet. In the treatment of flat foot he is extremely clear and thorough.

In speaking of operations in general, Whitman is often indefinite and one must refer to a general surgery to find exact direction as to methods. The discussion of apparatus is scattered and a special short chapter on the general principles involved would be a welcome addition. The book, altogether, is very satisfactory and practical, and the illustrations, type, and general appearance most pleasing.

P. Le B.

